# Syphilitic Eruptions

**Published:** 1843-03-18

**Authors:** 


### *Academy of Medicine, Paris.*

#### SYPHILITIC ERUPTIONS.

M. Jolly presented a report on a memoir by M. Gibert, on syphilitic eruptions.

The question proposed by the author is—are these diseases always secondary? Are they never primary? To answer it he examines all the doctrines and facts recorded on the subject, and concludes that syphylitic diseases of the skin are invariably, the consequence of a primary venereal infection.

Having combatted the ideas of M. Ricord, with respect to inoculation, the author proceeds to describe syphilitic eruptions, which he divides into eight species, distinguished by their external characters.

M. Gibert recommends a constitutional treatment, and gives a preference to mercurial preparations, in favour of which the best authorities are united.

In answer to the objections of M. Gibert, respecting inoculation, M. Ricord observed that he was about to present the academy an interesting preparation, but would first allude to a subject which so nearly interested him. To prevent repetition he would enounce the following propositions:—

No secondary symptoms, and consequently no syphilitic diseases of the skin, can occur without having been preceded by chancre.

If, to admit the existence of chancre, we require the presence of a certain number of external appearances—the characters denominated *Hunterian*—there is an end to all science on the subject. The specific character of chancre does not exist in the form, color, base of the ulcer, &c., but in the nature of the pus, which it secretes, and which may be determined by inoculation.

On the other hand, if the existence of chancres is to be denied unless the ulcer be seen, we are compelled to deny the possibility of their existence in situations where the eye cannot penetrate; in the urethra, for example, and in the vagina or neck of the uterus, before M. Ricord succeeded in introducing the use of the speculm as a means of diagnosis in these disorders. It must be remarked, that the occurrence of chancre of the urethra is rare in proportion to the distance of the part attacked from the meatus urinarius. The question of the bubo *d'embleé* which, at first sight, seemed to contradict the doctrine of M. Ricord, only confirms it.

This species of bubo never contains pus capable of being inoculated; or, if the pus be of this nature, it will be found that the bubo was always preceded by chancre, either apparent or masked. An interesting fact recently occurred confirmatory of this assertion. An *interne* of the Venereal Hospital had a bubo which he thought to be one of this species, and as the pus discharged by it was not inoculable, he considered the fact irreconcilable with M. Ricord's theory. On examining the urethra, however, carefully, he found a deep-seated chancre within the orifice of the urethra.

After these few remarks M. Ricord showed the preparation already alluded to. It was taken from a patient at the Venereal Hospital, who laboured under a gonorrhœa which resisted every species of treatment. A fistulous abscess opened in the perineum. The patient was afterwards attacked by the phthisis and died. The lungs were filled with tubercles, and the whole of the canal of the urethra contained numerous ulcers of a tubercular nature. The prostate gland, also, contained a great number of tubercles.—*Lond. Prov. Med. Journ.* Jan. 14.

